# Infecção por COVID-19 em Transplante de Coração: Relatos de Caso

**DOI:** 10.36660/abc.20200554

**Published:** 2020-09-18

**Authors:** Ligia Espinoso Schtruk, Jacqueline Miranda, Vitor Salles, Ana Sales, Luciana Lobbe, Vaisnava Cavalcante, Elisangela Reis, Sharon Kugel, Bruno Marques, Gabrielle Carvalho, Ruth Maia, Filipe Oliveira dos Reis, Danielle Rodrigues

**Affiliations:** 1 Instituto Nacional de Cardiologia Rio de Janeiro RJ Brasil Instituto Nacional de Cardiologia - Serviço de Insuficiência Cardíaca e Transplante, Rio de Janeiro, RJ - Brasil

**Keywords:** Doença de Chagas, Transplante do Coração, Imunossupressão, COVID-19/complicações, Comorbidades

No final de 2019, a infecção por um novo coronavírus, causando síndrome respiratória aguda grave, foi relatada em Wuhan, na China.^1^ Em 11/03/2020, a COVID-19 foi caracterizada pela Organização Mundial da Saúde como uma pandemia.^2^ O Brasil notificou o primeiro caso na América Latina em 26/02/2020.^2^ Considerando o curto período de tempo desde o início da pandemia, foram poucos os casos relatados de infecção em receptores de transplante cardíaco^3^. Pouco se sabe sobre as manifestações clínicas e a evolução do quadro neste grupo de pacientes.

## Relatos de Caso

Caso 1: sexo masculino, 47 anos, branco, portador de doença de Chagas, transplante cardíaco em 08/08/2012, sem comorbidades, em uso de tacrolimus (4 mg/dia), micofenolato de sódio (1.440 mg/dia).

**Figura 1:**
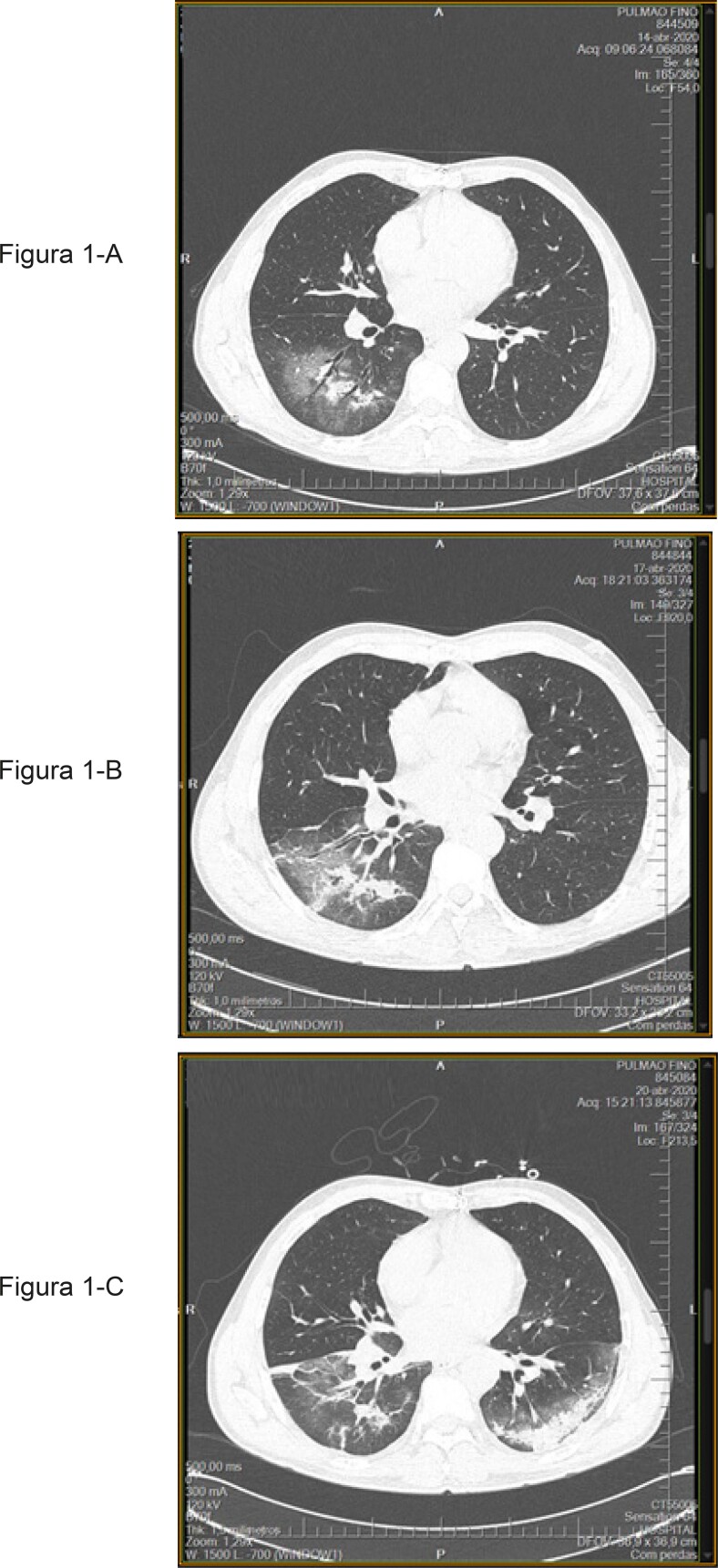
– Caso 1- Tomografia Computadorizada de Tórax.

Em 12/04/2020, apresentou mialgia, febre, cefaleia, dispneia leve, tosse seca, diarreia e anosmia. Ele mora em uma comunidade com casos confirmados de COVID-19. Em 14/04/2020 (D3 dos sintomas) procurou atendimento de urgência. Exame físico: febril (T. axilar: 38ºC), sem instabilidade hemodinâmica (PA: 100 x 60mmHg, FC: 60 bpm), saturação limítrofe de oxigênio (SpO_2_: 93%). A gasometria arterial mostrou hipóxia leve. Os demais exames são apresentados na Tabela 1. A tomografia computadorizada (TC) de tórax foi realizada na admissão, evidenciando imagem em vidro fosco bilateral, com distribuição multilobar, predominantemente periférica, com padrão de pavimentação em mosaico, associada a focos e áreas de consolidação. localizado nos lobos inferiores, com envolvimento <25% (Figura 1-A). A secreção nasofaríngea foi coletada para o teste RT-PCR SARS-COV2, o qual foi positivo. O paciente foi hospitalizado. O micofenolato de sódio foi suspenso (trombocitopenia / leucopenia), o tacrolimus foi mantido e a prescrição de anticoagulação profilática iniciada. No 3º dia de internação (D6 dos sintomas), o paciente manteve-se clinicamente estável (94% SatO_2_), com febre e piora da imagem radiológica, com consolidação em hemitórax direito, e intensificação da opacificação em vidro fosco em hemitórax esquerdo (Figura 1-B). Azitromicina (500 mg/d - 5 dias) e Ceftriaxona (2 g/d - 7 dias) foram iniciados. No 6º dia de internação, o nível sérico de tacrolimus era de 4,3 ng/dL e a dose foi ajustada. No 6º dia de internação (D9 dos sintomas), a febre persistia. A tomografia computadorizada revelou progressão da doença, com comprometimento de <50% do parênquima pulmonar (Figura 1-B). Foi iniciada hidroxicloroquina (dose de 400 mg 12/12h 1ª / dia, 400 mg/dia- 4 dias). No 6º dia de hospitalização, a leucopenia/linfopenia/ trombocitopenia melhoraram e o micofenolato de sódio foi reintroduzido. Nessa data, um aumento nos níveis de D-dímero foi observado (<500 ug/L a 2.270 ug/L). O Doppler vascular de membros inferiores não mostrou alterações. Ele recebeu alta hospitalar no 12º dia de internação, encontrando-se assintomático. Os testes de laboratório são mostrados na Tabela 1.

**Tabela 1:** – Exames laboratoriais: hospitalização e alta hospitalar

Exames	Hospitalização		Alta hospitalar	
	Caso 1	Caso 2	Caso 1	Caso 2
Sat O_2_ (%)	95	94	95	97
Hemoglobina (g%)	14	15,2	14	15,3
Leucócitos (mm^3^)	1.670	7.890	9.220	7.480
Neutrófilos (mm^3^)	910	6.043	5.983	5.056
Linfócitos (mm^3^)	439	623	1825	1264
Plaquetas (mm^3^)	87.000	278.000	293.000	417.000
PCR*	2	19	0,7	1,6
Creatinina	1,07	1,04	0,95	1,16
Ferritina	400	1992	1217	2208
D-dímero (ug/L)	310	3.000	590	690
Tacrolimus (ng/dl)	9,9	8,63	5,6	7,75

* PCR: Proteína C-Reativa.

Caso 2: sexo masculino, 54 anos, branco, com cardiomiopatia dilatada idiopática, submetido a transplante cardíaco em 03/08/2012. Comorbidades: hipertensão arterial sistêmica e artrite gotosa. Ele recebeu tacrolimus (2 mg/dia) e micofenolato de mofetil (1000 mg/dia).

**Figura 2:**
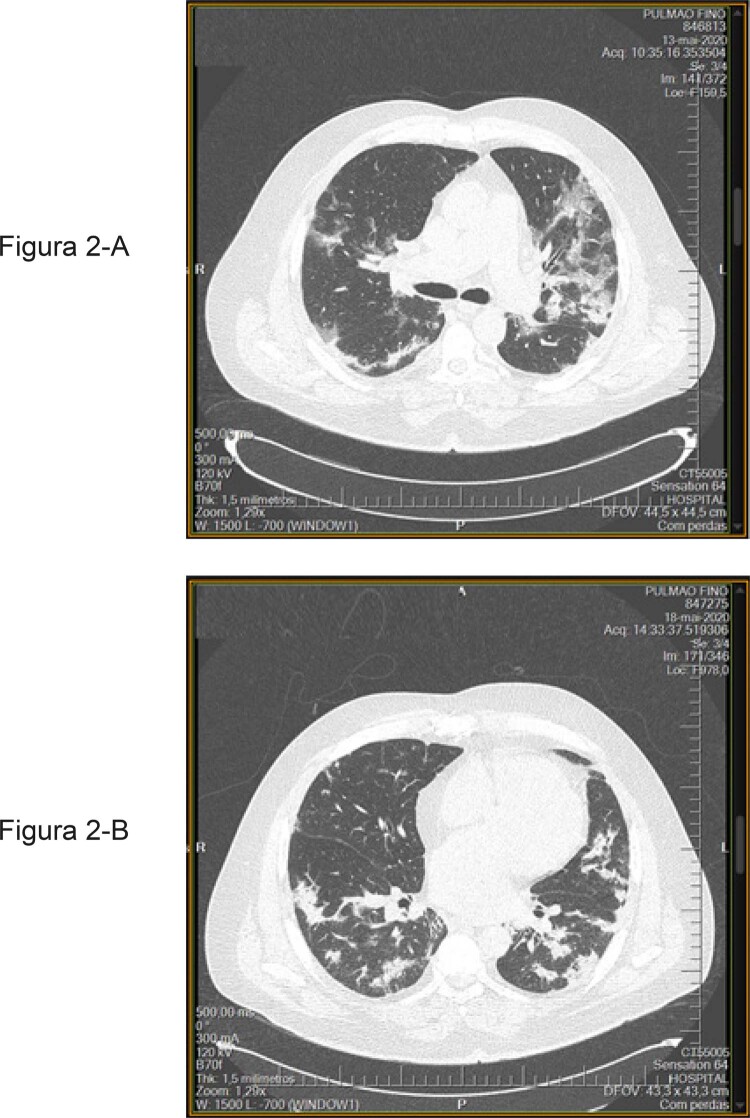
– Caso 2- Tomografia Computadorizada de Tórax.

No dia 11/05/2020 apresentou febre (38 ºC), tosse seca, falta de apetite e astenia. Sua filha tinha os mesmos sintomas. Em 13/05/2020 (D3 dos sintomas) procurou o pronto-socorro, e o exame físico não evidenciou febre ou instabilidade hemodinâmica (PA: 118x 78 mmHg, FC: 86 bpm); saturação de oxigênio (SpO_2_: 94%), sem dispneia. A gasometria arterial estava normal; os demais exames são apresentados na Tabela 1. A TC de tórax evidenciou áreas de atenuação em vidro fosco bilateralmente, com distribuição periférica predominante, com espessamento septal e alguns focos de consolidação associados, comprometimento <50% (Figura 2-A). A secreção nasofaríngea foi coletada para o teste RT-PCR SARS-COV2, o qual foi inconclusivo. O paciente foi hospitalizado. A imunossupressão foi mantida (sem leucopenia/ trombocitopenia). Os exames laboratoriais mostraram parâmetros inflamatórios elevados (Tabela 1) e níveis elevados de D-dímero. Foram prescritas ceftriaxona (2 g/dia- 7 dias), azitromicina (500 mg/dia- 5 dias) e enoxaparina (0,5 mg/kg /dia). No 3º dia de internação (D6 dos sintomas), encontrava-se clinicamente estável (95% SatO_2_), sendo o último dia com febre. O ecocardiograma mostrou função sistólica biventricular preservada (FE por Teichholz: 70,4). No 6º dia de hospitalização (D9 dos sintomas) a tomografia computadorizada evidenciou melhora da imagem radiográfica, acometendo menos de <50% do parênquima pulmonar (Figura 2-B). Em 18/05/20 uma segunda amostra de secreção nasofaríngea foi coletada para o teste de RT-PCR SARS-COV2, o qual permaneceu inconclusivo. O paciente apresentou melhora da linfopenia, redução dos parâmetros inflamatórios e queda progressiva dos níveis de D-dímero. Recebeu alta hospitalar no 10º dia de internação, encontrando-se assintomático. Os testes de laboratoriais são mostrados na Tabela 1.

## Discussão

A imunossupressão tem sido descrita como fator de risco de maior gravidade na infecção por COVID-19, assim como idade avançada, doenças cardiovasculares, diabetes mellitus, neoplasias e doenças pulmonares crônicas.^4^ Nesses casos, a apresentação clínica foi moderada e semelhante à relatada em pacientes não-imunossuprimidos.^4^ Os resultados inconclusivos do teste de RT-PCR SARS-COV2 de *swab* nasofaríngeo do Caso 2 estão de acordo com estudos que mostram que apenas 63% dos testes realizados foram positivos.^5^ Apesar de não haver critérios para hospitalização, de acordo com as recomendações da OMS e do Ministério da Saúde do Brasil, optou-se por indicar a internação, visto que as doenças virais respiratórias representam uma causa importante de morbimortalidade em pacientes imunocomprometidos.^1,6,7^ O caso 1 permaneceu febril até o 10º dia da doença, com leucopenia, linfopenia grave e trombocitopenia, o que nos obrigou a suspender o uso de micofenolato de sódio. Não foi necessário suspender o regime de imunossupressão no Caso 2. Apesar da imagem radiológica e dos marcadores inflamatórios, não houve piora ventilatória ou hipoxemia durante o curso da doença. As imagens tomográficas foram compatíveis com o diagnóstico e, no Caso 2, os parâmetros clínicos e radiológicos permitiram o diagnóstico.^8^ Estudos demonstram a participação imunológica na etiopatogênesis dos distúrbios da coagulação associados à COVID-19.^9^ Os pacientes apresentaram elevação do D-dímero, mas no Caso 1, esse aumento ocorreu mais tarde, no 11º dia de doença. Eles usaram profilaxia para trombose, e não houve eventos tromboembólicos. Atualmente, não há terapia específica comprovada disponível para COVID-19.^9-11^ O tratamento segue indicações semelhantes às dos pacientes não-transplantados. O regime de hidroxicloroquina foi utilizado com base na eficácia *in vitro* e dados clínicos sobre o tratamento para COVID-19 no primeiro paciente, mas não no segundo. Essa mudança ocorreu porque, nesta data, os estudos não mostraram evidências de grande eficácia desse medicamento no tratamento da COVID-19.^10,11^ Azitromicina e Ceftriaxona foram utilizadas devido a uma possível associação com pneumonia bacteriana.

Mudanças no regime imunossupressor devem ser analisadas individualmente, de acordo com a evolução de cada caso. A evolução clínica não foi mais grave do que a observada em pacientes não-imunossuprimidos. Estudos são necessários para avaliar se o uso de imunomoduladores poderia atenuar a cascata inflamatória. Nestes casos específicos, não foi observada inflamação exuberante, que poderia estar associada ao uso crônico de imunossupressores.
