# Supplementary material for: Infecção por COVID-19 em Transplante de Coração: Relatos de Caso
Source: Arq Bras Cardiol. 2020 Sep 18;115(3):574–8. [Article in Portuguese] doi: 10.36660/abc.20200554 (PMC9363096; doi:10.36660/abc.20200554)
Supplement: Translation

# Translation: COVID-19 Infection in Heart Transplantation: Case Reports

**Authors:** Ligia Espinoso Schtruk, Jacqueline Miranda, Vitor Salles, Ana Sales, Luciana Lobbe, Vaisnava Cavalcante, Elisangela Reis, Sharon Kugel, Bruno Marques, Gabrielle Carvalho, Ruth Maia, Filipe Oliveira dos Reis, Danielle Rodrigues

**Affiliations:** 1 Brazil Instituto Nacional de Cardiologia - Serviço de Insuficiência Cardíaca e Transplante, Rio de Janeiro, RJ - Brazil

**Keywords:** Chagas Disease, Heart Transplantation, Immunossupression, COVID-19/complications, Comorbidity

In late 2019, infection with a new coronavirus, causing severe acute respiratory syndrome, was reported in Wuhan-China.^1^ On 03/11/2020, COVID-19 was characterized by the World Health Organization as a pandemic.^2^ Brazil reported the first case in Latin America on 02/26/2020.^2^ Given the short time since the beginning of the pandemic, there have been few reported cases of infection in heart transplant recipients^3^. Little is known about the clinical manifestations and evolution of the condition in this group of patients

## Case Report

Case 1: 47-year-old male, Caucasian, with Chagas’ Disease, heart transplantation on 08/08/2012, without comorbidities, using tacrolimus (4 mg/day), sodium mycophenolate (1440 mg/day).

On 04/12/2020, he had myalgia, fever, headache, mild dyspnea, dry cough, diarrhea and anosmia. He lives in a community with confirmed cases of COVID-19. On 04/14/2020 (D3 of symptoms) he sought emergency care. Physical examination: feverish (axillary T: 38ºC), without hemodynamic instability (BP: 100 x 60 mmHg, HR: 60 bpm), borderline oxygen saturation (SpO_2_: 93%). Arterial blood gases showed mild hypoxia. Other exams are shown in Table 1. Chest computed tomography (CT) was performed on admission, and showed a bilateral ground-glass image, with a multilobar distribution, predominantly peripheral, with a crazy paving pattern, associated with foci and areas of consolidation, located in the lower lobes, <25% involvement (Figure 1-A). Nasopharyngeal secretion was collected for RT-PCR SARS-COV2 test, which was positive. The patient was hospitalized. Sodium mycophenolate was suspended (thrombocytopenia/leukopenia), tacrolimus was maintained, and prophylactic anticoagulation prescription was started. On the 3^rd^ day of hospitalization (D6 of symptoms), the patient remained clinically stable (94% SatO_2_), with fever and worsening of the radiological image, with consolidation in the right hemithorax, and intensification of the ground glass opacification in the left hemithorax (Figure 1-B). Azithromycin (500 mg/d - 5 days) and Ceftriaxone (2 g/day- 7 days) were started. On the 6^th^ day of hospitalization, the serum tacrolimus level was 4,3 ng/dL, and the dose was adjusted. On the 6^th^ day of hospitalization (D9 of symptoms), the fever persisted. A CT scan showed disease progression, with impairment of <50% of the lung parenchyma (Figure 1-B). Hydroxychloroquine was started (dose of 400 mg 12/12h 1^st^/day, 400mg/day- 4 days). On the 6^th^ day of hospitalization, leukopenia/lymphopenia/thrombocytopenia improved, and mycophenolate sodium was reintroduced. On this date, an increase in D-dimer levels was observed (<500 ug/L to 2.270 ug/L). Vascular Doppler of lower limbs did not show any alterations. He was discharged on the 12^th^ day of hospitalization, being asymptomatic. Laboratory tests are shown in Table 1.

**Table 1:** – Laboratory tests: admission and discharge

Exams	Admission		Discharge	
	Case 1	Case 2	Case 1	Case 2
Sat O_2_ (%)	95	94	95	97
Hemoglobin (g%)	14	15,2	14	15,3
Leukocytes (mm^3^)	1670	7890	9220	7480
Neutrophils (mm^3^)	910	6043	5983	5056
Lymphocytes(mm^3^)	439	623	1825	1264
Platelets (mm^3^)	87000	278000	293000	417000
CRP*	2	19	0,7	1,6
Creatinine	1,07	1,04	0,95	1,16
Ferritin	400	1992	1217	2208
D-dimer (ug/L)	310	3000	590	690
Tacrolimus (ng/dl)	9,9	8,63	5,6	7,75

* CRP: C-Reactive protein.

**Figure 1:** – Case 1- Computed Tomography of the Chest.

Case 2: 54-year-old male, Caucasian, with Dilated Idiopathic Cardiomyopathic, underwent heart transplantation on 08/03/2012. Comorbidities: systemic arterial hypertension and gouty arthritis. He received tacrolimus (2 mg/day) and mofetil mycophenolate (1000 mg/day).

On 05/11/2020 he had fever (38 ºC), dry cough, lack of appetite and asthenia. His daughter had the same symptoms. On 05/13/2020 (D3 of symptoms) he sought emergency care, and the physical examination showed no fever or hemodynamic instability (BP: 118x 78 mmHg, HR: 86 bpm); oxygen saturation (SpO_2_: 94%), without dyspnea. Arterial blood gases were normal; other exams are shown in Table 1. Chest CT showed bilateral ground-glass attenuation areas, with a predominant peripheral distribution, with septal thickening and some associated consolidation foci, <50% involvement (Figure 2 -A). Nasopharyngeal secretion was collected for RT PCR SARS-COV2 test, which was inconclusive. The patient was hospitalized. Immunosuppression was maintained (no leukopenia/thrombocytopenia). The laboratory exams showed high inflammatory parameters (Table 1) and high D-dimer levels. Ceftriaxone (2 g/day- 7 days), Azithromycin (500 mg/day- 5 days), and enoxaparin (0.5 mg/kg/day) were prescribed. On the 3^rd^ day of hospitalization (D6 of symptoms), he was clinically stable, (95% SatO_2_), and that was the last day he had fever. The echocardiogram showed preserved biventricular systolic function (Teichholz EF: 70.4). On the 6^th^ day of hospitalization (D9 of symptoms) the CT scan showed improved radiographic image, affecting less than <50% of the lung parenchyma (Figure 2-B). On 05/18/20 a second nasopharyngeal secretion was collected for RT-PCR SARS-COV2 test, which remained inconclusive. The patient showed improvement in the lymphopenia, reduction of inflammatory parameters and progressive drop in D-dimer levels. He was discharged on the 10^th^ day of hospitalization, being asymptomatic. Laboratory tests are shown in Table 1.

**Figure 2:** – Case 2- Computed Tomography of the Chest.

## Discussion

Immunosuppression has been described as a risk factor for greater severity in COVID-19 infection, as well as advanced age, cardiovascular diseases, diabetes mellitus, neoplasms and chronic lung diseases.^4^ In these cases, the clinical presentation was moderate and similar to that reported in non-immunosuppressed patients.^4^ The inconclusive results of the RT-PCR SARS-COV2 test of the nasopharyngeal swab in Case 2 is in agreement with studies showing that only 63% of the tests performed were positive.^5^ Although there are no criteria for hospitalization, according to the recommendations from the WHO and the Brazilian Ministry of Health, we chose to indicate hospitalization, as respiratory viral diseases represent a significant cause of morbidity and mortality in immunocompromised patients.^1,6,7^ Case 1 remained feverish until the 10^th^ day of illness, with leukopenia, severe lymphopenia and thrombocytopenia, which made us suspend the use of mycophenolate sodium. It was not necessary to suspend the immunosuppressive regimen of Case 2. Despite the radiological image and inflammatory markers, there was no ventilatory worsening or hypoxemia during the course of the disease. Tomographic images were compatible with the diagnosis, and in Case 2, clinical and radiological parameters allowed the diagnosis to be attained.^8^ Studies demonstrate immunological participation in the etiopathogenesis of coagulation disorders associated with COVID-19.^9^ The patients showed D-dimer elevation, but in Case 1 this increase occurred later, on the 11^th^ day of illness. They used prophylaxis for thrombosis, and there were no thromboembolic events. There is currently no proven specific therapy available for COVID-19.^9-11^ The treatment follows indications similar to that in non-transplant patients. The hydroxychloroquine regimen was used based on *in vitro* effectiveness and clinical data on treatment for COVID-19 in the first patient, but not in the second. This change occurred because on this date, studies have not shown evidence of great effectiveness of this drug in the treatment of COVID-19.^10,11^ Azithromycin and Ceftriaxone were used for the possible association with bacterial pneumonia.

Changes in the immunosuppressive regimen must be analyzed individually, according to the evolution of each case. The clinical evolution was not more severe than that observed in non-immunosuppressed patients. Studies are needed to assess whether the use of immunomodulators could mitigate the inflammatory cascade. In these specific cases, no exuberant inflammation was observed, which could be associated with the chronic use of immunosuppressants.
